# Incubation with a Complex Orange Essential Oil Leads to Evolved Mutants with Increased Resistance and Tolerance

**DOI:** 10.3390/ph13090239

**Published:** 2020-09-09

**Authors:** Daniel Berdejo, Elisa Pagán, Natalia Merino, Rafael Pagán, Diego García-Gonzalo

**Affiliations:** Departamento de Producción Animal y Ciencia de los Alimentos, Facultad de Veterinaria, Instituto Agroalimentario de Aragón-IA2 (Universidad de Zaragoza-CITA), C/ Miguel Servet, 177, 50013 Zaragoza, Spain; berdejo@unizar.es (D.B.); epagan@unizar.es (E.P.); merino@unizar.es (N.M.); pagan@unizar.es (R.P.)

**Keywords:** complex orange essential oil, *Staphylococcus aureus*, genotypic resistance, mutagenesis frequency, whole genome sequencing, minimum inhibitory and bactericidal concentrations, growth kinetics, antibiotic susceptibility

## Abstract

Emergence of strains with increased resistance/tolerance to natural antimicrobials was evidenced after cyclic exposure to carvacrol, citral, and (+)-limonene oxide. However, no previous studies have reported the development of resistance and tolerance to complex essential oils (EOs). This study seeks to evaluate the occurrence of *Staphylococcus aureus* strains resistant and tolerant to a complex orange essential oil (OEO) after prolonged cyclic treatments at low concentrations. Phenotypic characterization of evolved strains revealed an increase of minimum inhibitory and bactericidal concentration for OEO, a better growth fitness in presence of OEO, and an enhanced survival to lethal treatments, compared to wild-type strain. However, no significant differences (*p* > 0.05) in cross-resistance to antibiotics were observed. Mutations in *hepT* and *accA* in evolved strains highlight the important role of oxidative stress in the cell response to OEO, as well as the relevance of the cell membrane in the cell response to these natural antimicrobials. This study demonstrates the emergence of *S. aureus* strains that are resistant and tolerant to EO (*Citrus sinensis*). This phenomenon should be taken into account to assure the efficacy of natural antimicrobials in the design of food preservation strategies, in cleaning and disinfection protocols, and in clinical applications against resistant bacteria.

## 1. Introduction

Essential oils (EOs) and their individual constituents (ICs) have been proposed as food preservatives [1] and as disinfection agents due to their antimicrobial properties [2] and better social acceptance as compared to chemically synthesized compounds [3]. Moreover, since it is commonly accepted that these natural antimicrobials, they do not induce mutations that could lead to antimicrobial resistance (AMR) or tolerance [4]; they have also been studied for the treatment of bacterial infections in order to prevent the emergence of resistances to antibiotics [5,6,7]. Several authors have reported that exposure to ICs or EOs during bacterial growth maintains or even reduces the mutation rate [8,9,10]. This fact could be explained due to the antioxidant activity of EOs and ICs at low doses [11], which decrease the formation of reactive oxygen species (ROS) and, consequently, bacterial mutagenesis [12]. However, recent studies have demonstrated that cyclic exposure to ICs can lead to the emergence of strains that are resistant and tolerant to carvacrol, citral, or (+)-limonene oxide, since random mutations occurring in the bacterial population can provide a greater degree of fitness or survival than wild-type strain [9,10,13]. In addition, some of these strains showed increased resistance and tolerance to other natural antimicrobials, to antibiotics, and even to other methods of food preservation or disinfection such as heat or pulsed electric fields [9]. According to Balaban, et al. [14], resistance is the ability of bacteria to replicate in the presence of an antimicrobial, usually at low doses for long periods of time, while tolerance is the bacterial capacity to survive at lethal doses of the antimicrobial.

Complex essential oil of sweet orange (OEO) is one of the most widely used EOs on an industrial level [15], widely employed in different fields such as food, cosmetics, pharmaceutics, and agrochemicals [16,17,18]. This EO is obtained from the peels of *Citrus sinensis* (L.) by cold pressing and is composed of more than 20 ICs, including limonene (>85%), myrcene, α-pinene, and sabinene [19,20,21]. The excellent antimicrobial properties of OEO can be attributed to that complex composition of ICs, both because of the functional groups that each IC presents and because of the synergism that occurs between them [21,22]. Several authors point out that cell envelopes are one of the most important bacterial targets of ICs and therefore of EOs, along with the internal damage caused by the accumulation of ROS when the antimicrobial is applied at high doses [23,24,25]. The emergence of EO-resistant or EO-tolerant strains has hitherto been ruled out due to the multitude of antimicrobial action mechanisms that EOs can exert on bacteria in view of their great complexity and compositional variety [26]. In order to develop resistance mechanisms to complex EOs, bacteria would have to mutate genes involved in multiple structures or metabolic pathways. Actually, no studies have evidenced as yet the emergence of EO-resistant or EO-tolerant strains through evolution assays [27,28,29].

In genomic studies of strains evolved in the presence of ICs [9,10,13], several mutations were related to general cell response mechanisms. For instance, mutations in *soxR,* a redox-sensitive transcriptional regulator, were related to carvacrol resistance and tolerance in *Escherichia coli* [30] and *Salmonella enterica* [13]. Evolved strains of *Staphylococcus aureus* likewise displayed an increased resistance and tolerance to carvacrol: not due to improved intrinsic antimicrobial resistance, but rather to a better repair of cellular damage [10]. Hence, if these mutations appeared in the presence of complex EOs, they would probably enhance general resistance and tolerance mechanisms, or provide an improved system for repairing cell damage, thereby leading to the emergence of resistances or tolerances to EOs. Moreover, the identification of genetic variations responsible for increased resistance or tolerance would allow for a better grasp of the mechanisms of action of natural antimicrobials (as yet not completely understood), and thus facilitate the design of more effective IC and EO treatments, as well as combating the emergence of AMR.

Therefore, the objectives of this study are a) to determine the emergence of *Staphylococcus aureus* strains resistant and tolerant to a complex essential oil of orange (*Citrus sinensis*) by evolution assays, b) to evaluate the direct resistance and tolerance of the evolved strains against orange essential oil, as well as cross-resistance to antibiotics, and c) to identify genetic modifications occurring during the evolution assay which lead to increased resistance/tolerance.

## 2. Results

### 2.1. Isolation of Resistant Strains by Evolution Assay with OEO 

After evolution assay (20 days or steps), five colonies from a plate were randomly selected, namely, SaROEO_1–5_ (i.e., SaROEO_1_, SaROEO_2_, SaROEO_3_, SaROEO_4_, and SaROEO_5_), to carry out phenotypic characterization and to evaluate the emergence of resistant strains. Firstly, the antimicrobial resistance and tolerance of SaWT and SaROEO_1–5_ against OEO was evaluated by testing minimum inhibitory concentration (MIC) and minimum bactericidal concentration (MBC), respectively (Table 1). 

Since MIC and MBC values for the 5 isolated colonies were similar (*p* > 0.05), results are shown for the group SaROEO_1–5_. MIC results revealed a >200% increased resistance of SaROEO_1–5_ to OEO in comparison with SaWT. An OEO concentration of 1500 µL/L was enough to inhibit growth of SaWT, while SaROEO_1–5_ could still grow in the presence of 5000 µL/L of OEO. Similarly, MBC data demonstrated the increased tolerance of the evolved strains to OEO: MBC was increased >100%, from 2500 µL/L for SaWT to >5000 µL/L for SaROEO_1–5_. It was not possible to determine MIC and MBC values above 5000 µL/L for SaROEO_1–5_ due to OEO solubility problems and the high resistance and tolerance displayed by those evolved strains.

MIC and MBC results revealed that all the colonies of the evolution assay displayed the same degree of resistance and tolerance to OEO. These results suggest that all isolated colonies were identical, and that the bacterial cultures obtained from the evolution assay were probably homogeneous. We therefore selected one of the five evolved strains for further experiments, from here onward referred to as SaROEO.

### 2.2. SaROEO Showed a Greater Fitness than SaWT in Presence of OEO

**Figure 1:**
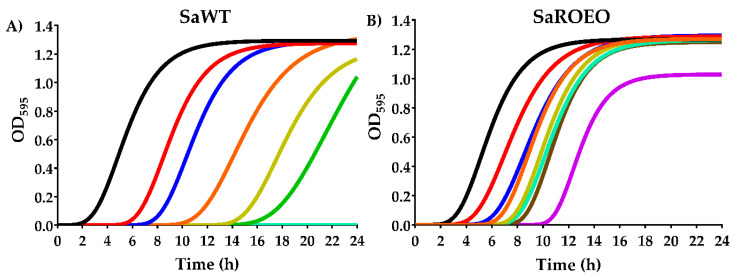
Growth curves of *Staphylococcus aureus* USA300 (**A**; SaWT) and evolved strain (**B**; SaROEO) in the absence (▬) and presence of 250 (▬), 500 (▬), 750 (▬), 1000 (▬), 1250 (▬), 1500 (▬), 2000 (▬), 5000 (▬) of orange essential oil (OEO), modelled using the modified Gompertz equation (Equation (1)).

In order to further study the resistance of SaROEO, growth kinetics in tryptone soya broth (TSBYE) were studied at different concentrations of OEO. First, growth curves were obtained in absence and presence of OEO for SaWT and SaROEO, and modelled (Figure 1) by modified Gompertz equation (Equation (1)). In agreement with MIC results, OEO concentrations higher than or equal to 1500 µL/L did not allow the growth of SaWT, while SaROEO could reach the stationary phase in presence of 5000 µL/L OEO at 18 h of growth (Figure 1).

Table 2 summarizes the parameters of the modified Gompertz equation: *A* (maximum OD_595_), *µ_m_* (maximum specific growth rate) and *λ* (lag phase time), for both strains under all the conditions tested. The standard error, *R^2^* and *R^2^* adjusted values, and the root mean square error (*RMSE*) supported a good least-squares adjustment for both strains under all the concentrations tested (Table S1). Firstly, the growth parameters revealed that the presence of OEO slows down the microbial growth of both strains. Specifically, as the concentration of OEO was increased, *µ_m_* and *A* were slightly reduced (*p* ≤ 0.05), and *λ* was intensely prolonged (*p* ≤ 0.05). Comparing the evolved strain with SaWT, with regard to *A*, a similar behaviour (*p* > 0.05) was shown at OEO concentrations below the MIC of SaWT (≤1500 µL/L), hovering *A* values around 1.19−1.32 OD_595_. No significant differences (*p* > 0.05) were observed in the *µ_m_* between SaWT and SaROEO at OEO concentrations of 500 µL/L or lower. At higher concentrations, SaROEO showed a higher *µ_m_*, than SaWT (*p* ≤ 0.05). For instance, the *µ_m_* of SaWT was reduced from 0.248 OD_595_/h (without OEO) to 0.167 OD_595_/h in presence of 1250 µL/L of OEO, while growth rate of SaROEO was not modified (*p* > 0.05) at this concentration (0.240 OD_595_/h) compared to strains evolved in the absence of OEO (control).

The major differences between SaWT and SaROEO were found in *λ* at all the tested OEO concentrations, but more prominently at high concentrations; the lag phase lasted a total of 17.4 h for SaWT at 1250 µL/L, thus 10 h longer than the SaROEO lag phase at the same OEO concentration.

### 2.3. Higher Survival of SaROEO after OEO Treatments at both pH 7.0 and 4.0

**Figure 2:**
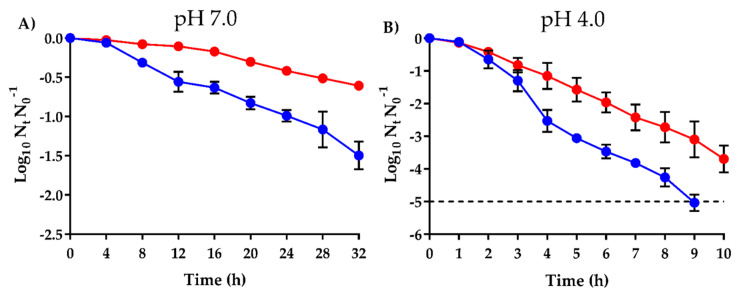
Survival curves of *Staphylococcus aureus* USA300 (●; SaWT) and evolved strain (●; SaROEO), after 2000 µL/L orange essential oil (OEO) treatment at pH 7.0 (**A**) and pH 4.0 (**B**) at 37 °C. Data are means ± standard deviations (error bars) obtained from at least 3 independents experiments. Dashed line represents the detection limit (−5.0 log_10_).

In order to further evaluate the tolerance of SaROEO, survival curves were obtained after lethal treatment with 2000 µL/L of OEO at pH 7.0 and pH 4.0 and compared to those of SaWT (Figure 2). These pH values were chosen as representative of neutral and acid conditions within the usual pH range of foods [31].

At neutral pH, significant differences (*p* ≤ 0.05) were observed between SaWT and SaROEO inactivation after lethal treatments of OEO. While SaWT showed a bacterial reduction of 1.5 log_10_ cycles after 32 h of treatment at pH 7.0, only 0.6 log_10_ cycles of SaROEO were inactivated (Figure 2A). At pH 4.0 (Figure 2B), the inactivation reached was greater in both strains than at neutral pH. Similarly, SaROEO also exhibited a higher survival to the lethal treatment at acid pH compared to SaWT. For instance, after 9 h of treatment at pH 4.0, more than five log_10_ cycles of SaWT population were inactivated, whereas just over three log_10_ cycles of inactivation were achieved for SaROEO.

### 2.4. SaROEO Displayed an Antibiotic Resistance Similar to SaWT

Finally, a disk diffusion test was carried out to evaluate cross-resistance against antibiotics. Table 3 presents the inhibition halos of SaWT and SaROEO against tetracycline, chloramphenicol, nalidixic acid, rifampicin, norfloxacin, novobiocin, trimethoprim, and cephalexin.

Inhibition halos larger than 15 mm were obtained for the antibiotics tested in order to be able to evaluate variations in the resistance of SaROEO compared to SaWT. It should be noted that *S. aureus* USA300 is a methicillin-resistant strain. Susceptibility assay revealed that none of the tested antibiotics featured significant differences (*p >* 0.05) between SaWT and SaROEO in terms of cross-resistance. Thus, the mutations that occurred during the evolution assay would not be related with resistance against a wide range of antibiotics: tetracyclines, quinolones, and aminoglycosides.

### 2.5. OEO does not Induce an Increased Mutagenesis

**Figure 3:**
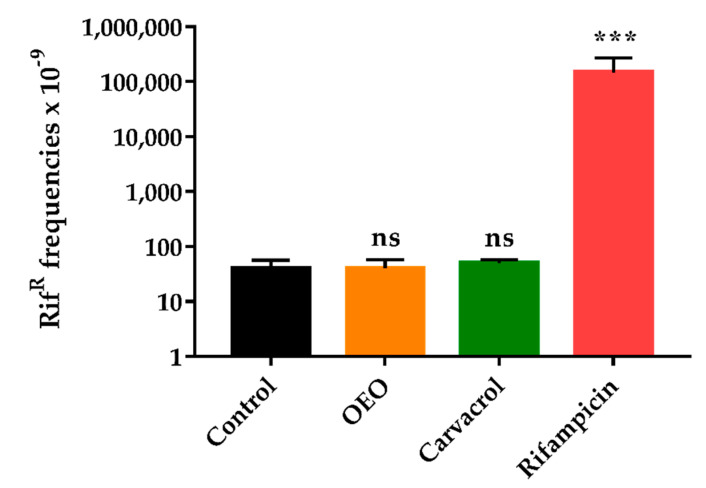
Mutagenesis frequency in *S. aureus* USA300 grown in broth (control, ■) and with orange essential oil (OEO; 750 µL/L; ■), carvacrol (50 µL/L, ■) and rifampicin (0.01 mg/L, ■). Mutagenesis frequency was expressed as rifampicin-resistant cells in the total microbial population. Data are means ± standard deviations (error bars) obtained from five independent experiments. ns: no statistically significant differences (*p* > 0.05); ***: statistically significant differences (*p* ≤ 0.001), in comparison with control.

The mutation frequency was determined for SaWT in the absence or in the presence of OEO (at the same concentration used in the evolution assay, i.e., 1/2 MIC) to evaluate whether this complex EO could increase the mutation rate, which, in turn, would facilitate the emergence of genotypic resistances [32]. As shown in Figure 3, SaWT displayed a spontaneous frequency of rifampicin-resistant mutants over 60 × 10^−9^ during bacterial growth in absence of the OEO (control). Similar results were obtained when OEO and carvacrol were added to growth medium. A *t*-test revealed no significant differences (*p* > 0.05) among the control, the OEO at 750 µL/L, and carvacrol at 50 µL/L.

On the contrary, the presence of the rifampicin at 0.01 mg/L (1/2 MIC for SaWT) in the growth medium led to a mutation frequency around 150 × 10^−6^; thus 1000 times higher compared to control or when natural compounds (OEO or carvacrol) were added.

### 2.6. Four Missense Mutations Identified in SaROEO

**Figure 4:**
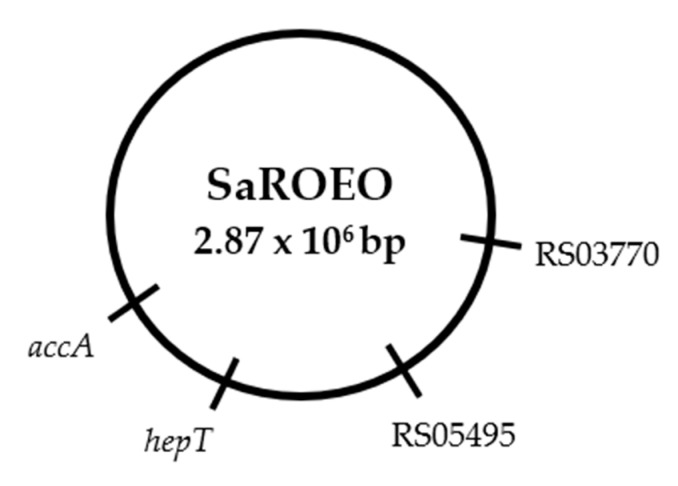
Genomic map of evolved strain (SaROEO) in comparison with *Staphylococcus aureus* USA300 (SaWT).

Whole genome sequencing (WGS) was performed on SaROEO and compared to SaWT genome in order to identify the mutations causing the increased resistance to OEO that occurred during the evolution assay. A total of 17.31 and 4.19 million of 150 bp-reads were obtained for SaWT and SaROEO, respectively. From those reads, 90.52% and the 88.05% displayed a Phred quality score above 30. The quality-control-filtered paired-end reads were mapped at 91.60% and 96.44%, respectively, on the reference genome sequence of *S. aureus* USA300 (NCBI accession: NC_007793.1). The reference genome was sufficiently covered to allow the detection of genetic variations between the strains studied; an at least 100-fold coverage depth was achieved for both strains. This study focused on the genetic variations between SaWT and SaROEO in order to identify the mutations which occurred during the evolution assay. After WGS, Sanger sequencing verified a total of four single nucleotide variations (SNVs) in the comparison of genomic sequence of SaROEO with that SaWT (Figure 4).

In addition, these mutations were also confirmed by Sanger sequencing in all the strains isolated in the evolution assay (SaROEO_1–5_), supporting the supposition of homogeneity of the bacterial population at the end of the evolution assay.

Table 4 summarizes the genes involved in the mutations, as well the proteins coded in order to ascertain the cause of the increased resistance and tolerance to OEO observed in SaROEO.

The four mutations detected in SaROEO were the following:

(1) A SNV was detected at position 993 bp in the SAUSA300_RS03770 locus resulting in a change of glutamic acid by asparagine in the enzyme allophanate hydrolase at position 331 amino acid.
(2) A transversion from thymine to guanine was found at position 26 bp in SAUSA300_RS05495 locus coding a hypothetical protein in *S. aureus* USA300.
(3) A replacement of cytosine by thymine was observed at position 272 bp in the *hepT* gene. This missense mutation resulted in a protein modification in the position 91 amino acid, from threonine to isoleucine, in the heptaprenyl diphosphate synthase subunit II.
(4) A transition from cytosine to thymine was detected at position 481 bp in the *accA* gene. This mutation led to a protein change in the position 161 of proline to serine in the acetyl-CoA carboxylase carboxyl transferase subunit alpha.

## 3. Discussion

Cyclic exposure to prolonged subinhibitory doses of OEO enabled the selection of evolved strains of *S. aureus*: SaROEO_1–5._ MIC and MBC results revealed the increased resistance and tolerance to the OEO of *S. aureus* after the evolution assays. All five isolated strains SaROEO_1–5_ showed a >200% increased resistance to OEO in comparison with SaWT (Table 1). Similarly, a >100% increase in MBC of the isolated strains revealed their higher tolerance compared to SaWT. It should be noted that the phenotypic characterization was performed with fresh cultures grown in the absence of OEO, thereby supporting the supposition that the increase in resistance and tolerance is stable and based on genetic modifications in the evolved strain. In addition, pending sequencing results, the same increase in resistance and tolerance to OEO displayed by the five isolated colonies (SaROEO_1–5_) would imply that the same mutations were fixed in the microbial population, and therefore the culture obtained after the evolution assay would be homogeneous. Despite the development of resistance in *S. aureus* against antibiotics [33] and against other antimicrobial compounds such as peptides [34], no resistance against EO has been previously described to the best of our knowledge. These results provided the first evidence that the evolution protocol had led to the occurrence of resistant strains against a complex EO. Following the same evolution assay for 10 days against ICs, Berdejo, et al. [10] reported an increase in MIC values in *S. aureus* USA300 from 50% to 100% against carvacrol, citral, and (+)-limonene oxide, compared to wild-type strain. An increase in direct resistance to ICs has also been observed in Gram-negative bacteria after evolution assays; similar increases have also been observed in *S. enterica* (between 50% and 100%), and even greater in *E. coli* (up to 300%) in comparison with wild-type strain.

The study of growth kinetics in the presence of the OEO revealed significant differences (*p* < 0.05) in growth fitness between SaWT and SaROEO (Figure 1). Increases in OEO concentration led to a decrease in the maximum value of OD_595_ (*A*), which means that the bacterial concentration reached was lower when OEO concentration was higher. The addition of OEO to the growth medium also caused a reduction of the maximum specific growth rate (*µ_m_*), and a longer lag phase time (*λ*). These results support the assumption that both strains grow more slowly in the presence of the OEO, and that they need a longer time to adapt to the environment as the OEO concentration increases. It is likely that EO is disturbing the cell membrane integrity and increasing the membrane permeability of both strains, thereby leading to a prolonged adaptation and lag phase time [35]. Comparing SaROEO to SaWT, no significant differences (*p* < 0.05) were observed when strains were grown in absence of OEO. However, SaROEO showed a divergent growth behavior in presence of OEO: its *µ_m_* was greater and its *λ* shorter than for SaWT, and the differences became more pronounced as the concentration of OEO increased. These results not only support the assumption that SaROEO can grow at higher concentrations, but also indicate that its growth rate at low concentrations is much more pronounced than that of SaWT. In regard to growth kinetics of evolved strains in presence of natural antimicrobials, previous studies have also observed differences in growth models compared to wild-type strain, mainly in growth rate and lag phase. *S. enterica* carvacrol-resistant mutants exhibited a maximum specific growth rate 10-fold higher and a lag phase 7 h shorter than wild-type strain in presence of carvacrol at 150 µL/L [13]. Although differences were observed in both parameters, SaROEO results revealed a greater relevance of lag phase in the bacterial adaptation to OEO than in the adaptation of *S. enterica* to carvacrol. One of the main bacterial targets of EO is the cell membrane [35]; it is likely that mutations in SaROEO are related to the cell envelopes, which allow for a faster adaptation to OEO and consequently a decrease in lag phase time. These results would thus explain why mutations of SaROEO were fixed in the bacterial population after evolution assay (750 µL/L OEO). In addition, the emergence of resistant mutant strains could pose a risk in food preservation, because bacterial growth at low doses of OEO might be underestimated.

Survival curves obtained after OEO lethal treatments also showed an increased tolerance of SaROEO at both neutral (Figure 2A) and acid pH (Figure 2B) compared to SaWT. For instance, inactivation of SaWT was 2 log_10_ cycles higher than that of SaROEO after 9 h of OEO lethal treatment at pH 4.0. Similarly, IC-evolved strains of *E. coli* [9], *S. aureus* [10], and *S. enterica* [13] also showed an increase in direct tolerance. Previous studies have reported that EO could serve as an effective disinfectant agent to inactivate *S. aureus* [36] or to eradicate resistant forms, such as biofilms [36,37]. However, the presence of resistant strains might diminish the efficacy of disinfectants at the doses previously established for these purposes; the survival of such strains would pose a microbiological risk.

Development of mutant resistance after evolution assays with ICs indicated the occurrence of cross-resistance to antibiotics [13,30]. Carvacrol resistance developed by strains of *E. coli* [9] and *S. enterica* [13] likewise increased their resistance to tetracyclines, quinolones, aminoglycosides, and β-lactams. Those strains were mutated in genes related to stress response and to transcriptional regulators of sensor redox-cycling drugs, such as *soxR*, which are also involved in common bacterial resistance to all antibiotics tested [13,30]. However, in our study, SaROEO and SaWT (Table 3) displayed a susceptibility similar (*p* > 0.05) to that of all the tested antibiotics: tetracyclines, quinolones, and aminoglycosides. It should be noted that *S. aureus* USA300 is a methicillin-resistant strain. It is thus likely that the mutations which occurred in SaROEO during the evolution assay would not be related to general mechanisms of bacterial resistance, but rather to a specific resistance to OEO. In this regard, the clinical use of EO to combat bacterial infections and prevent AMR needs to be reconsidered [6,38].

Previous studies have demonstrated that EOs and ICs do not increase the frequency of mutation in bacteria [7,10]. Decreased mutation rates have actually been observed compared to controls, and they have been attributed to the antioxidant capacity of these natural compounds [11]. In accordance with our results, previous studies have reported a low mutation rate of *S. aureus* when it is exposed to natural compounds during growth, both in the presence of ICs [10] and EOs [8]. Our results, in accordance with those studies, show a mutagenesis frequency of SaWT that is similar both in absence or in presence of OEO at 1/2 MIC concentration (Figure 3). In contrast to antibiotics [32], the emergence of *S. aureus* mutants would not be caused directly by OEO, but because of the selective pressure of OEO on the growing bacterial population. In other words, OEO would select spontaneous and random mutants that emerge during evolution assay, and which display a better growth fitness than SaWT in presence of OEO. Finally, the selective pressure exerted by OEO would lead to the fixation of those growth-enhancing mutations in the bacterial population.

WGS and Sanger sequencing revealed four mutations in SaROEO in comparison to SaWT (Table 4). Given these mutations arose during the evolution assay, it is likely that some or all contribute to the cause of the increased resistance and tolerance observed in the phenotypic characterization of SaROEO. Consequently, this increase in resistance/tolerance allowed it to be isolated after the evolution assay at subinhibitory doses of OEO. Notably, these mutations were also verified in the five isolated strains (SaROEO_1–5_) stemming from the same evolution lineage, thereby demonstrating that at the end of the evolution assay the bacterial population was homogeneous, i.e., mutations had been fixed in the bacterial culture.

A missense mutation was found in SAUSA300_RS03770 locus, whose product is the allophanate hydrolase. This enzyme catalyzes the hydrolysis of allophanate to ammonium (NH_4_^+^) and carbon dioxide (CO_2_) [39], and belongs to the amidase signature family, a large group of hydrolytic enzymes that catalyse the hydrolysis of amide bonds (CO-NH_2_) [40]. In prokaryotes, this enzyme has only been related to bacterial metabolism [41,42]. However, Juttukonda, et al. [43] reported that allophanate synthase was related to resistance in *Acinetobacter baumannii* against calprotectin, a protein with chelating properties on divalent zinc and manganese metal ions. To the best of our knowledge, however, no studies have related the antimicrobial effect of EOs to the presence of those divalent metal ions.

A SNV was located in SAUSA300_RS05495 locus that encodes the DUF2129 domain. This is an uncharacterized domain found in various hypothetical prokaryotic proteins whose function has not been determined in vivo. Structural modelling suggests that this domain may bind nucleic acids [44]. However, due to the lack of information regarding this locus’s function, it is unknown how this mutation can influence the behavior of SaROEO.

The *hepT* gene mutation led to a change from threonine to isoleucine in the heptaprenyl diphosphate synthase subunit II. This enzyme is involved in the synthesis of menaquinone, also known as vitamin K_2_ [45]. It serves as a key electron transporter in many types of bacteria and is required for bacterial respiration where ROS are produced. According to Chueca, et al. [24,46], ROS are involved in bacterial death by several ICs, such as carvacrol, citral, and limonene: in fact, the latter is the main IC present in OEO. It is likely that the alteration of this metabolic pathway would cause a decrease in ROS accumulation, consequently resulting in a greater degree of survival to OEO. Evolved strains with increased resistance and tolerance to carvacrol also showed mutations in transcriptional regulators induced by oxidative stress, such as *soxR* and *yfhP*, in *E. coli* [30] and *S. enterica* [10]. Oxidative stress was also induced by treatment with complex EO in *Klebsiella pneumoniae* [47]. These results highlight the relevance of oxidative stress in the cell response to natural antimicrobials. Although SaROEO did not show increased cross-resistance to any antibiotics, Berti, et al. [48] reported that a SNV in the *hepT* gene was associated with a slight resistance increase and a pronounced increased tolerance to daptomycin.

Finally, a SNV was detected in *accA* in SaROEO. This gene codifies acetyl-CoA carboxylase carboxyl transferase subunit alpha, an enzyme involved in the malonyl-CoA biosynthesis pathway, which takes part in the formation of fatty acids [49]. Because fatty acids are constituents of membrane building phospholipids, this pathway is essential for bacterial growth [50]. In fact, carboxyl transferase subunits have been shown to be targets for antibiotic development [51]. According to Meades, et al. [52], cinnamon EO inhibits the carboxyltransferase subunit of *E. coli*, which partially explains that EO’s antibacterial activity. Moreover, as is well known, cell membranes are the first barrier and one of the main structures affected by natural antimicrobials, which alter their permeability and disrupt their integrity [35,47]. It is likely that the mutation of *accA* could result in improved cell membrane formation and/or repair in presence of OEO. This hypothesis would explain the increase of the resistance and tolerance of SaROEO, as well as its better adaptation to OEO in the growth curves. These findings support the relevance of fatty acid synthesis in the cell response to complex OEO.

Briefly, the two mutations identified in *hepT* and *accA* could explain the increased resistance and tolerance observed in the phenotypic characterization of SaROEO. These results highlight the likely relevance of oxidative stress response in the cell defense against OEO, along with the important role played by the membrane in the resistance and tolerance to it. It should be noted that the genetic variations described were located in the genome, not in mobile genetic elements such as plasmids, transposons, etc. Such mutations would thus be considered hereditable, leading to stable increased resistance and tolerance. Once the emergence of resistant strains against complex EO has been demonstrated, we recommend to follow Sullivan, et al. [53] and Tyers, et al. [54] in combining different antimicrobials with various cell targets in order to avoid the occurrence of resistant strains while improving their antimicrobial properties. For instance, ICs from OEO, such as limonene, myrcene, and sabinene, can be used in combination with first-line tuberculostatic antibiotics for the treatment of resistant *Mycobacterium tuberculosis*.

It should be noted that the emergence of strains resistant and tolerant to OEO could be due to the fact that its ICs have the same cell targets. To avoid the emergence of resistant and tolerant strains, further research is thus required to better understand the mode of action of natural antimicrobials in order to design optimal treatments that combine EOs and ICs with different functional groups, or even with other kinds of antimicrobials.

## 4. Materials and Methods

### 4.1. Microorganisms and Growth Conditions

The wild-type microorganism was *S. aureus* USA300 methicillin-resistant strain (FPR3757 strain), provided by Prof. Kolter laboratory (Harvard Medical School, Boston, MA, USA).

OEO used in this investigation was kindly provided by Indulleida S.A. (Lleida, Spain). This commercial EO was prepared using a mixture of different *Citrus sinensis* varieties (‘Washington Navel’, ‘Navelate’, ‘Navelina’, ‘Salustiana’, and ‘Valencia Late’) by cold press system extraction. The peels of fresh fruits were cold-pressed, the EO was separated from the crude extract by centrifugation, and stored in the dark in sealed glass vials at 4 °C until use. The composition of this batch of OEO (95.1% limonene, 2.0% myrcene, 0.7% α-pinene, 0.7% sabinene, and 1.5% other compounds) was previously analyzed by Bento, et al. [20].

Throughout this investigation, the strain was kept in cryovials at −80 °C with glycerol (20% *v*/*v*), from which plates of tryptone soya agar (Oxoid, Basingstoke, England) with 0.6% yeast extract (Oxoid; TSAYE) were prepared on a weekly basis. To prepare the working bacterial cultures, test tubes containing 5 mL of tryptone soya broth (Oxoid) with 0.6% yeast extract (TSBYE) were inoculated with one colony and then incubated aerobically on an orbital shaker (130 rpm; Heidolph Vibramax 100, Schwaback, Germany) for 12 h at 37 °C (Incubig, Selecta, Barcelona, Spain). Subsequently, flasks containing 10 mL of fresh TSBYE were inoculated with the resulting subculture to an initial concentration of 10^6^ colony forming units per mL (CFU/mL) and incubated for 24 h at 37 °C and 130 rpm until the stationary growth phase was reached (2 × 10^9^ CFU/mL approximately).

### 4.2. Minimum Inhibitory Concentration (MIC)

Minimum inhibitory concentration (MIC) of OEO was determined by inoculating the bacteria in test tubes with 5 mL of Mueller–Hinton broth cation adjusted (Sigma-Aldrich, Steinheim, Westphalia, Germany; MHB) at an initial concentration of 5 × 10^5^ CFU/mL in presence of different concentrations of OEO: from 250 up to 5000 µL/L, and incubated at 37 °C for 24 h and 130 rpm. This protocol was adapted from standard methods for antimicrobial susceptibility tests [55]. A vigorous shaking by vortex (Genius 3, Ika, Königswinter, Germany) was used to prepare OEO dispersions in MHB, avoiding the use of solvents for their potentially detrimental effect on antibacterial activity. Positive control tubes with 5 mL MHB inoculated at 5 × 10^5^ CFU/mL without OEO, and negative control tubes with 5 mL MHB were also prepared in each experiment. Once tubes were incubated, MIC was determined as the lowest concentration of the antimicrobial compound that was capable of avoiding bacterial growth. To objectively determine bacterial growth, optical density was read at 595 nm (OD_595_) using a microplate reader (Genios, Tecan, Männedorf, Switzerland). The values of OD_595_ were subtracted from the negative control with the same concentration of the oil as the sample (without bacterial inoculation), corresponding to the absorbance caused by the growth medium and the oil. In total, 10% of the OD_595_ measure of the positive control was established as the lowest limit to consider that bacterial strain had been grown [12].

### 4.3. Minimum Bactericidal Concentration (MBC)

The minimum bactericidal concentration (MBC) of OEO was evaluated from the test tubes employed in the MIC determination after incubation. 100 µL aliquot of each tube was spread onto Mueller–Hintonagar cation adjusted (Sigma-Aldrich; MHA) plates and incubated at 37 °C for 24 h. Colonies were counted and the lowest concentration of carvacrol that killed ≥99.9% of the initial bacterial concentration (5 × 10^5^ CFU/mL) was defined as the MBC end point [56]. The same positive and negative MIC test controls were employed in this experiment.

### 4.4. Evolution Assay of OEO

The evolution assay was based on the isolation of strains by prolonged exposure to subinhibitory concentration of OEO during bacterial growth [9,10,13]. *S. aureus* wild-type strain (SaWT) was grown on TSAYE plates for 24 h at 37 °C. A single colony was inoculated in 5 mL TSBYE and incubated under agitation for 12 h at 37 °C. This preculture was diluted 1:1000 into 50 mL TSBYE and incubated for 5.0 h to obtain an exponential growth phase culture. From this culture, SaWT were inoculated at an initial bacterial concentration of 10^6^ CFU/mL in 5 mL TSBYE with 750 µL/L of OEO (1/2 MIC). This bacterial suspension was incubated 24 h/37 °C/130 rpm and, once stationary phase was reached, the same dilution steps were repeated 20 times: the culture was inoculated (10^6^ CFU/mL) in 5 mL TSBYE with 750 µL/L of OEO and incubated 24 h/37 °C/130 rpm. After the 20th step, an aliquot was diluted in phosphate buffered saline (Sigma-Aldrich; PBS) and spread on TSAYE plates. After the incubation period, five evolved strains were randomly selected for phenotypic and genotypic characterization.

### 4.5. Growth Curves in Presence of OEO

Bacterial growth curves were obtained at different concentrations of OEO in TSBYE. Based on the results obtained in MIC assay, the concentration ranges of OEO used were 0–1500 μL/L for SaWT and 0–5000 μL/L for evolved strains. OEO was added in test tubes with 5 mL of TSBYE, vigorously vortexed, inoculated with the microbial culture (5 × 10^5^ CFU/mL), and incubated at 37 °C and 130 rpm for 24 h. During the culture incubation, OD_595_ of the test tubes was measured every hour in a microplate reader. A positive control (without antimicrobial added) and a negative control (without microbial culture added) were incorporated in all the assays. The values of OD_595_ obtained during the experiment were subtracted from the initial OD_595_ (at time 0), corresponding to the absorbance caused by the growth medium. Bacterial growth curves based on OD_595_ were graphically displayed and modelled by modified Gompertz equation (Equation (1)) [57].

(1) $$y=A\exp\left\{-exp\left[\;\left({µ}_{m}\;e/A\;\right)\left(\lambda -t\right)+1\;\right]\right\}$$

where *y*: OD_595_; *t*: time (h); *A*: maximum OD_595_ value reached; *µ_m_*: maximum specific growth rate (h^−1^); *λ*: lag phase time (h).

A least-squares adjustment was carried out to build the model and obtain A, µ_m_, and λ values using Prism 5.0 (GraphPad Software, Inc., San Diego, CA, USA). The adjustment’s goodness of fit was evaluated using standard error, *R*^2^ and *R*^2^ adjusted values, and the root mean square error (RMSE).

### 4.6. Survival Curves after Lethal OEO Treatments

Tolerance to OEO was studied by evaluating bacterial survival after lethal treatments. The treatment medium was citrate-phosphate McIlvaine buffer, prepared from citric acid monohydrate (Panreac) and disodium hydrogen phosphate (Panreac), adjusted to pH 7.0 and pH 4.0. These pH values were chosen as representative of neutral and acid conditions within the normal pH range of food. The treatment was carried out in 10 mL McIlvaine buffer at 37 °C, to which OEO was added at a concentration of 2000 µL/L and then vigorously vortexed. Stationary phase culture was centrifuged for 5 min at 6000 RCF in a microcentrifuge (Mini Spin, Eppendorf, Hamburg, Germany) and resuspended in the treatment medium at 10^7^ CFU/mL in order to initiate the lethal OEO treatment. During treatment, aliquots were obtained at established times. Those samples were adequately diluted in PBS and spread on TSAYE plates. After plates incubation (24 h/37 °C), colonies were counted by an automatic plate counter (Analytical Measuring Systems, Protos, Cambridge, United Kingdom). The increase in tolerance to OEO was evaluated by comparison of inactivation kinetics (i.e., survival curves) between SaWT and its evolved strains.

### 4.7. Antibiotic Susceptibility Test

Agar disk diffusion assay was used to test antimicrobial susceptibility according to the Clinical and Laboratory Standards Institute [55,58]. First, bacterial suspension was spread on MHA plates and, after 5 min at room temperature, blank disks (Thermo Scientific™ Oxoid™ Anti-microbial Susceptibility Disk Dispenser, ST6090, Waltham, MA, USA) were placed on the surface of plates and individually impregnated with 10 μL of each antibiotic: 30 µg tetracycline, 30 µg chloramphenicol, 400 µg nalidixic acid sodium, 50 µg rifampicin, 60 µg norfloxacin, 50 µg novobiocin sodium, 10 µg trimethoprim, and 150 µg cephalexin (Sigma-Aldrich). Plates were incubated at 37 °C for 16–18 h, after which the diameters of the resulting inhibition zones were measured (paper disks included).

### 4.8. Mutagenesis Frequency Evaluation

The mutagenic effect of OEO was determined by calculating the rate of mutants resistant to rifampicin due to point mutations in the *rpoB* gene [10,59]. Overnight culture of SaWT was diluted 1:10,000 into 50 mL TSBYE and incubated at 37 °C and 130 rpm for 24 h. This culture was then diluted 1:3 in tubes containing 10 mL TSBYE with 750 µL/L of OEO (same concentration used in the evolution assay) and without OEO. This experiment was also carried out with carvacrol (Sigma-Aldrich) at 50 µL/L as control of natural compounds from previous study [10] and with rifampicin (Sigma-Aldrich) at 0.01 mg/L concentration as a positive control of mutagenesis. Those suspensions were grown at 37 °C and 130 rpm for 24 h (ca. 2 × 10^9^ CFU/mL). Samples of the culture were serially diluted in PBS and pour-plated on TSAYE in the presence and absence of 100 mg/L rifampicin (Sigma-Aldrich). Plates were incubated at 37 °C for 48 h and colonies were counted. Mutation rates were calculated by dividing the number of colonies present in rifampicin plates (mutation events) by the number of colonies present in plates without antibiotic [60].

### 4.9. Whole Genome Sequencing (WGS) and Identification of Genetic Variations

Genomic DNA (gDNA) was extracted using a gDNA kit (DNeasy kit, Qiagen, Hilden, Germany) from bacterial strains. Illumina technology was used to carry out whole genome sequencing (WGS) on NextSeq equipment at mid output flow, with a total of 2 × 150 cycles (Illumina; Fasteris, SA, Geneva, Switzerland). Subsequently, quality control was performed with FastQC software (version 0.11.9, https://www.bioinformatics.babraham.ac.uk/projects/fastqc/) evaluating the quality of the reads (Q_30_), sequence length, presence of adapters, and overrepresented and duplicated sequences. The quality control-filtered paired-end reads were mapped on the reference genome sequence (NCBI accession: NC_007793.1): *S. aureus* subsp. *aureus* USA300_FPR3757 [61], using a Burrows–Wheeler Alignment (BWA) tool (version 0.7.5a) [62] and Samtools software (version 1.2) [63]. A raw 100-fold depth coverage was achieved for both strains. Samtools was then applied to remove potential PCR duplicates according to reading positions on the reference genome; the resulting BAM files were then further processed using LoFreq-Star (version 2.1.1, source: http://csb5.github.io/lofreq/) to correct mapping errors and insert the quality values. Finally, single nucleotide variants (SNV) and short insertions (Ins) and deletions (Dels) were detected using LoFreq-Star, and toolbox snpEff (source: http;//snpeff.sourceforge.net/) was employed to identify involved genes and to predict functional effect variations [64]. Coverage was further analyzed by the Integrative Genomics Viewer (IGV; Broad Institute, version 2.8.9, source: https://software.broadinstitute.org/software/igv/) in order to find structural variations (SVs). Although mapping was carried out against the reference genome, SNVs, Ins, Dels, and SVs were identified between our wild type and evolved strains to determine the mutations which had occurred during the evolution assay. The resulting genome sequences were deposited in the Sequence Read Archive (SRA) of NCBI (Bioproject ID: PRJNA657166). The accession numbers of the samples are SAMN15817977 (SaWT) and SAMN15817978 (SaROEO). Finally, specific primers (Table S2) were designed to carry out PCR amplifications for Sanger sequencing to verify the mutations detected in the WGS.

### 4.10. Statistical Analysis

All phenotypic characterization results (MIC and MBC determination, growth curves, lethal treatments, and antibiotic susceptibility test) were obtained from at least three independent experiments carried out on different working days with different bacterial cultures. Growth curve parameters, lethal treatment graphics, and antibiotic susceptibility tests are displayed as the mean ± standard deviation, using Prism 5.0. Data were analyzed and submitted to comparison of averages using analysis of variance (ANOVA), followed by post-hoc Tukey test and *t*-tests with Prism 5.0, and differences were considered significant if *p* ≤ 0.05.

## 5. Conclusions

This study demonstrates for the first time the emergence of resistant and tolerant strains of *S. aureus* against a complex essential oil (EO) (*Citrus sinensis*). Prolonged exposition of *S. aureus* to low doses of sweet orange EO (OEO) led to the emergence of resistant strains (SaROEO). SaROEO displayed an increase of >200% in resistance and >100% in tolerance to OEO, compared to SaWT, by MIC and MBC determination, respectively. Moreover, SaROEO showed a better growth fitness in presence of OEO and a greater degree of survival to lethal treatments at both acid and neutral pH. WGS of SaROEO allow us to identify the genetic variations that occurred during the evolution assay responsible for that strain’s increased resistance to OEO. Among the four mutations verified by Sanger sequencing, two were located in the genes *hepT* and *accA*. These genes highlight the important role of oxidative stress in the cell response to complex EO, as well as the relevance of the cell membrane in the resistance and tolerance against these natural antimicrobials. Nevertheless, it is certainly possible that mutations located in genes codifying other mechanisms and structures would give rise to yet undiscovered resistances similar to these.

These results highlight the great importance of taking these resistances into account, since evolved strains could represent a microbiological risk due to their ability to grow and survive under conditions established for their corresponding wild-type strains. Consequently, in order to ensure the efficacy of natural antimicrobials, the emergence of resistant strains should be taken into account in the design of food preservation strategies, or in cleaning and disinfection protocols. In this regard, further research will be fundamental in defining how such strains resistant and tolerant to natural antimicrobials emerge. Likewise, it is key to better understand the mechanisms of bacterial inactivation of EOs and ICs in order to enhance their antimicrobial properties as a food preservative, as cleaning and disinfection agents, or even in their potential clinical use against multi-drug resistant bacteria.

## Figures and Tables

**Table 1:** Minimum inhibitory concentration (MIC; µL/L) and minimum bactericidal concentration (MBC; µL/L) of orange essential oil (OEO) for *Staphylococcus aureus* USA300 (SaWT) and evolved strain (SaROEO_1–5_, 5 isolated strains). Each value represents the result of 5 different experiments carried out for every strain tested, with different bacterial cultures and on different working days.

Strains	Carvacrol Concentration (µL/L)												
**SaWT**	0	250	500	750	1000	1250	1500	1750	2000	2500	3000	4000	5000
**SaROEO_1–5_**	0	250	500	750	1000	1250	1500	1750	2000	2500	3000	4000	5000

■ Cell growth; ■ Neither cell growth nor inactivation; ■ Cell inactivation.

**Table 2:** *A* (maximum OD_595_), *µ_m_* (maximum specific growth rate) and *λ* (lag phase time) parameters of the modified Gompertz model obtained from growth curves of *Staphylococcus aureus* USA300 (SaWT) and evolved strain (SaROEO), at different concentrations of orange essential oil (OEO). Each value represents the mean ± standard deviation from 3 independent experiments.

OEO (µL/L)	*A* (OD_595_)		*µ_m_* (OD_595_/h)		*λ* (h)	
	SaWT	SaROEO	SaWT	SaROEO	SaWT	SaROEO
**0**	1.294 ± 0.012 ^a^	1.268 ± 0.010 ^A^	0.248 ± 0.019 ^a^	0.254 ± 0.018 ^A^	2.902 ± 0.228 ^a^	3.319 ± 0.191 ^A^
**250**	1.276 ± 0.014 ^a^	1.287 ± 0.013 ^A^	0.238 ± 0.015 ^a^	0.217 ± 0.014 ^AB^	6.468 ± 0.215 ^b^	4.776 ± 0.215 ^B^*
**500**	1.293 ± 0.013 ^a^	1.295 ± 0.019 ^A^	0.229 ± 0.011 ^a^	0.218 ± 0.015 ^AB^	8.239 ± 0.162 ^c^	6.311 ± 0.251 ^C^*
**750**	1.324 ± 0.030 ^ab^	1.271 ± 0.018 ^A^	0.181 ± 0.008 ^b^	0.244 ± 0.021 ^AB^*	11.410 ± 0.170 ^d^	6.876 ± 0.271^D^*
**1000**	1.262 ± 0.032 ^ab^	1.280 ± 0.018 ^A^	0.183 ± 0.006 ^b^	0.251 ± 0.020 ^A^*	15.000 ± 0.099 ^e^	7.884 ± 0.243 ^E^*
**1250**	1.185 ± 0.038 ^b^	1.280 ± 0.017 ^A^	0.167 ± 0.008 ^b^	0.240 ± 0.017 ^AB^*	17.430 ± 0.149 ^f^	7.761 ± 0.226 ^F^*
**1500**	/	1.272 ± 0.019 ^A^	/	0.210 ± 0.014 ^AB^	/	8.062 ± 0.237 ^G^
**1750**	/	1.254 ± 0.019 ^AB^	/	0.237 ± 0.019 ^AB^	/	8.447 ± 0.236 ^H^
**2000**	/	1.246 ± 0.018 ^ABC^	/	0.244 ± 0.019 ^AB^	/	8.563 ± 0.225 ^I^
**2500**	/	1.212 ± 0.023 ^BC^	/	0.250 ± 0.019 ^A^	/	9.482 ± 0.240 ^J^
**3000**	/	1.198 ± 0.019 ^C^	/	0.227 ± 0.017 ^AB^	/	9.516 ± 0.204 ^K^
**4000**	/	1.112 ± 0.020 ^D^	/	0.208 ± 0.015 ^AB^	/	10.300 ± 0.196 ^L^
**5000**	/	1.052 ± 0.014 ^E^	/	0.193 ± 0.010 ^B^	/	10.850 ± 0.139 ^M^

Different superscript letters represent statistically significant differences (*p* ≤ 0.05) among the means of the same column; * Significantly different from SaWT (*p* ≤ 0.05); /: no growth.

**Table 3:** Zones of growth inhibition (mm) for agar disk diffusion assays of *Staphylococcus aureus* USA300 (SaWT) and evolved strains (SaROEO) against antibiotics: 30 µg tetracycline, 30 µg chloramphenicol, 400 µg nalidixic acid sodium, 50 µg rifampicin, 60 µg norfloxacin, 50 µg novobiocin sodium, 10 µg trimethoprim and 150 µg cephalexin. Each value represents the mean diameter of the inhibition halo ± standard deviation from three independent experiments.

Antibiotics	Strains	
	SaWT	SaROEO
Tetracycline	28.07 ± 1.11	28.93 ± 2.03
Chloramphenicol	22.23 ± 1.32	23.37 ± 1.25
Nalidixic acid	15.88 ± 1.24	15.45 ± 1.27
Rifampicin	30.80 ± 0.67	30.51 ± 0.26
Norfloxacin	11.60 ± 0.31	12.02 ± 0.73
Novobiocin	27.38 ± 1.24	28.39 ± 1.56
Trimethoprim	22.18 ± 1.05	20.39 ± 1.20
Cephalexin	14.45 ± 0.72	13.39 ± 1.23

**Table 4:** Mutations of evolved strain (SaROEO) in comparison with *Staphylococcus aureus* USA300 (SaWT). Single nucleotide variation (SNV).

Genome Position	Gene	Locus Tag ^a^ (Old Locus Tag)	Mutation ^b^	Change	Information
**776,659**	*-*	RS03770 (0702)	SNV: A993T	Glu331Asp	Allophanate hydrolase
**1,118,342**	*-*	RS05495 (1021)	SNV: T26G	Ile9Ser	Hypothetical protein (DUF2129 domain containing protein)
**1,526,963**	*hepT*	RS07410 (1359)	SNV: C272T	Thr91Ile	heptaprenyl diphosphate synthase subunit II
**1,808,243**	*accA*	RS08985 (1646)	SNV: C481T	Pro161Ser	Acetyl-CoA carboxylase carboxyl transferase subunit alpha

**^a^** The gene locus tag corresponds to SAUSA300_RSXXXXX. Also, old locus tag is provided SAUSA300_XXXX; **^b^** Position respect to the start of the coding region.

## Supplementary Materials

The following are available online at https://www.mdpi.com/1424-8247/13/9/239/s1. Table S1: *A* (maximum OD595), *µm* (maximum specific growth rate; h-1) and *λ* (lag time; h) values and error standard of the modified Gompertz model obtained from 3 independently growth curves of *Staphylococcus aureus* subsp. *aureus* USA300_FPR3757 (SaWT) (A) and SaROEO at different concentrations of OEO, Table S2: Primers used for PCR amplification and Sanger sequencing to verify the mutations in SaROEO.
