# Supplementary material for: Incubation with a Complex Orange Essential Oil Leads to Evolved Mutants with Increased Resistance and Tolerance
Source: Pharmaceuticals (Basel). 2020 Sep 9;13(9):239. doi: 10.3390/ph13090239 (PMC7557841; doi:10.3390/ph13090239)
Supplement: Supplementary file 1 [file pharmaceuticals-13-00239-s001.pdf]

Table S1. A (maximum OD_{595}), μ_m (maximum specific growth rate; h^{-1}) and λ (lag time; h) values and error standard of the modified Gompertz model obtained from 3 independently growth curves of *Staphylococcus aureus* subsp. *aureus* USA300_FPR3757 (SaWT) (A) and SaROEO at different concentrations of OEO. The goodness of the fit is shown by R^2 and adjusted R^2 values and the root mean square error (*RMSE*).

A) SaWT

OEO (µL/L)	Values			Standard error			Goodness of fit		
	A	μ_{max}	λ	A	μ_{max}	λ	R^2	Adj. R^2	*RMSE*
**0**	1.294	0.248	2.902	0.012	0.019	0.228	0.9841	0.9832	0.0550
**250**	1.276	0.238	6.468	0.014	0.015	0.215	0.9897	0.9891	0.0517
**500**	1.293	0.229	8.239	0.013	0.011	0.162	0.9942	0.9938	0.0407
**750**	1.324	0.181	11.410	0.030	0.008	0.170	0.9920	0.9916	0.0470
**1000**	1.262	0.183	15.000	0.032	0.006	0.099	0.9964	0.9961	0.0251
**1250**	1.185	0.161	17.430	0.038	0.008	0.149	0.9893	0.9887	0.0295

B) SaROEO

OEO (µL/L)	Values			Standard error			Goodness of fit		
	A	μ_{max}	λ	A	μ_{max}	λ	R^2	Adj. R^2	*RMSE*
**0**	1.268	0.254	3.319	0.010	0.018	0.191	0.9888	0.9881	0.0465
**250**	1.287	0.217	4.776	0.013	0.014	0.215	0.9895	0.9889	0.0494
**500**	1.295	0.218	6.311	0.019	0.015	0.251	0.9871	0.9863	0.0576
**750**	1.271	0.244	6.876	0.018	0.021	0.271	0.9835	0.9825	0.0660
**1000**	1.280	0.2518	7.884	0.018	0.020	0.243	0.9862	0.9854	0.0614
**1250**	1.280	0.240	7.761	0.017	0.017	0.226	0.9888	0.9881	0.0555
**1500**	1.272	0.210	8.062	0.019	0.014	0.237	0.9882	0.9875	0.0564
**1750**	1.254	0.237	8.447	0.019	0.019	0.236	0.9858	0.9850	0.0622
**2000**	1.246	0.244	8.563	0.018	0.019	0.225	0.9850	0.9842	0.0636
**2500**	1.212	0.250	9.482	0.023	0.019	0.240	0.9848	0.9839	0.0624
**3000**	1.198	0.227	9.516	0.019	0.017	0.204	0.9868	0.9860	0.0581
**4000**	1.112	0.208	10.300	0.020	0.015	0.196	0.9893	0.9887	0.0485
**5000**	1.052	0.193	10.850	0.014	0.010	0.139	0.9953	0.9950	0.0316

Table S2. Primers used for PCR amplification and Sanger sequencing to verify the mutations in SaROEO.

SaROEO mutations	Forward primer (5' → 3')	Reverse primer (5' → 3')
SAUSA300_RS03770	ATCGCATGGGGATGATGCTT	TGGGCTACAACTCATATTTGCTT
SAUSA300_RS05495	GCACCGACTAGTACCGCATT	GTACAGCCCCCTCAAGCATT
hepT	AAATCACTTCCGACCGGCTT	CGTAGTTTTACAGGGGGCGT
accA	ACCGCCAAGTGGTTCAGAAA	TTGCGCGTTTGCAAGAAAGA
